# The Control of the Expansion or Compression of Colloidal Crystals Lattice with Salt Solution

**DOI:** 10.3390/nano14040355

**Published:** 2024-02-13

**Authors:** Hongwei Zhou, Wenze Ouyang, Shuangyang Zou, Shenghua Xu

**Affiliations:** 1 Key Laboratory of Microgravity, Institute of Mechanics, Chinese Academy of Sciences, Beijing 100190, China; zhouhongwei@imech.ac.cn (H.Z.); oywz@imech.ac.cn (W.O.); shuangyang.zou@imech.ac.cn (S.Z.); 2 School of Engineering Science, University of Chinese Academy of Sciences, Beijing 100049, China

**Keywords:** colloidal crystals, lattice spacing, diffusiophoresis, diffusioosmosis

## Abstract

Tuning the lattice spacing or stop bands holds great significance in the design and application of materials with colloidal crystals. Typically, particle surface modifications or the application of external physical fields are needed. In this study, we demonstrated the ability to expand or compress the lattice of colloidal crystals simply by utilizing a salt solution, without the need for any special treatments to the colloidal particles. We found that by only considering the diffusiophoresis effect we cannot explain the reversion of lattice expansion to lattice compression with the increase in the salt concentration and that the diffusioosmotic flow originating from the container wall must be taken into account. Further analysis revealed that variations in the salt concentration altered the relative amplitudes between diffusiophoresis and diffusioosmosis through changing the zeta potentials of the particles and the wall, and the competition between the particle diffusiophoresis and wall diffusioosmosis lay at the center of the underlying mechanism.

## 1. Introduction

Colloid crystals or photonic crystals, formed by nanoparticles in the periodic order, are a very promising and intriguing material because of their versatile application in high-performance sensors, novel display devices, optical fibers, and eco-friendly paintings [1,2,3,4,5]. The optical properties of colloid crystals, such as the photonic stop band or structural color, are primarily modulated by the lattice spacing, refractive index, and orientation. Therefore, various approaches, including the application of external electric and magnetic fields [6,7,8,9], mechanical stress [10,11,12,13], and so on, have been pursued to accomplish the following objective. Although tremendous advances have been made in recent years, looking for a convenient and energy-efficient method to tune the lattice spacing of photonic crystals is still one of the focuses in the relevant studies.

Moreover, compared to photonic crystal films and photonic crystals immobilized in polymer gels, the colloidal crystals formed in the liquid phase as a result of repulsive interactions were different, according to the research community. Strategies to strengthen inter-particle interactions [14,15,16] or to enhance the particles’ response to external physical stimuli are usually necessary. Nevertheless, these adjustments may restrict their utilization in specific domains. In addition, once colloidal crystals have formed, altering their lattice spacing becomes arduous without external energy impartation. Therefore, seeking a general and convenient approach to attain an extensive range of lattice spacing modulation is highly desirable for colloidal crystals formed by untreated particles. Compared to other methods, the use of an electric field has intrinsic advantages, because colloidal particles are naturally charged. Nevertheless, in lower field strengths, the Coulomb forces exerted on particles are weak due to a large screening effect, leading to a limited tuning range. In higher field strengths, electrochemical side reactions, including water electrolysis, not only affect the performance of the electrode but also result in the releases of ions, which causes a significant decrease in reflection intensity [6,17,18,19]. In some cases, the colloidal crystals may even melt.

In an aqueous solution, even a slight concentration gradient of salt can lead to the autonomous and spontaneous migration of colloidal particles, which is a fascinating phenomenon referred to as the diffusiophoresis (DP) effect. This effect arises from the inherent surface charge of the colloidal particles and was initially observed by Derjaguin in capillary osmosis and film formation from latex depositions [20,21,22,23,24]. Recent investigations have already showed the utilization of diffusiophoresis and its associated diffusioosmosis (DO) effect to propel particles [25,26,27], as well as to separate and assemble particles [27,28,29,30,31]. In electrolyte-driven diffusiophoresis, both the zeta potentials of the particles and walls are crucial factors, although they are often assumed to remain constant under different experimental conditions for the sake of convenience in analysis. However, it should be noted that the zeta potential can vary significantly with the physical and chemical properties of the surrounding fluid, such as the ionic strength, pH, temperature, and so on [32,33,34,35,36,37,38,39]. According to the classical diffusiophoresis theory, the direction and velocity of the particles’ movement are directly dictated by the zeta potential. Therefore, the adjustability of the zeta potential presents an opportunity to control the migration of particles and, consequently, to modulate the lattice spacing of formed crystals.

In this study, we aim to demonstrate the control of the lattice expansion or compression of colloidal crystals by simply adjusting the salt concentration, without the need for particle surface modifications or an external energy input. This ability to tune the lattice spacing is analogous to the aforementioned method utilizing the electric field. Theoretical analysis reveals that the competition between particle diffusiophoresis and wall diffusioosmosis serves as the underlying mechanism. This opens up the possibility for further tuning of the stop band of colloidal crystals using diffusiophoresis.

## 2. Materials and Methods

### 2.1. Materials

The negatively charged polystyrene particles with sulfonic groups on their surface were purchased from Huge Biotechnology (Shanghai, China). The received particles were purified by repeated washing with ultrapure water (UPRLC5DRO UPW system, Relatec, Beijing, China) in the centrifugation process, and then stored with an ion-exchange resin (Amberlite IRN-150, EMD Millipore Corporation, Darmstadt, Germany) for further deionization.

The mean diameter and polydispersity of the particles determined by dynamic light scattering (Brookhaven, BI-200SM, Holtsville, NY, USA) were 90 nm and 6%, respectively. The effective transported charge of the particles was about 680, obtained by the number density of conductivity method. The zeta potentials of the particles dispersed in different salt concentrations (*C*_s_) were measured by Zeta PALS (Brookhaven, Holtsville, NY, USA). The streaming potentials of the tube wall were measured by SurPASS (Anton Paar, Graz, Austria) after splitting and compressing the tube into a flat sheet.

### 2.2. Sample Preparation and Experimental Procedure

The sample suspensions with particles of a volume fraction *Φ* = 1.2% were prepared by carefully mixing the particle suspension with a certain amount of ultrapure water, and then the mixtures were sonicated for several minutes to attain homogeneous ones. Finally, some resin was added for further deionization. The densities of the water and polystyrene colloidal particles were 1.0 g/cm^3^ and 1.05 g/cm^3^, respectively. The roughly matched densities and the small size of the particles ensured that the precipitation effect could be ignored.

**Figure 1:**
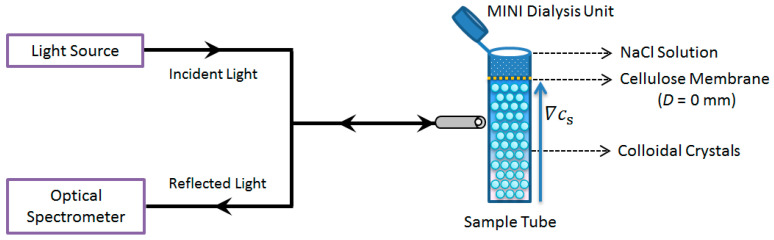
Sketch of the experimental setup.

The experimental setup is shown in Figure 1. On the left side of the sample tube, there is a typical scheme reflection spectroscopy, which contains a tungsten halogen light source (Avantes, Avalight-HAL, Apeldoorn, The Netherlands), a fiber optical spectrometer (Avantes, Avaspec-2048, Apeldoorn, The Netherlands), and a bifurcated fiber optic cable. The incident light was vertical to the wall of the sample tube, and the reflected light was detected by the spectrometer. The sample tube is a cylindrical quartz tube (with an inner diameter of 10 mm and a length of 30 mm) that is open at the upper end. At the top of the sample tube, there is a dialysis device (Slide-A-Lyzer MINI Dialysis Units, Thermo Fisher Scientific, Rockford, IL, USA), which was a hollow cylinder (outer diameter 9.5 mm, length 12 mm) made of polypropylene. The bottom of the unit was cellulose membrane (MWCO: 3500), and the top side of the unit had a cap as a seal.

A reflection spectroscopy was used to determine the crystal lattice type and the lattice constant of the colloidal crystals [40,41]. The wave vector of each reflection peak can be calculated by the following:

(1) q=(4πν/λ)sin⁡(θ/2)

where *λ* is the peak wavelength, θ (=180°) is the angle between the incident and reflected light, and *ν* is the refractive index of the medium, which can be estimated from the indices of the particle and the water using $\nu =\nu_{p}\phi +\nu_{w}\left(1-\phi \right)$. According to the ratio of the wave vector corresponding to each peak, the lattice type can be determined. Then, the lattice constant *L_a_* can be evaluated with the following:

(2) $$L_{a}=(\lambda /2\nu )\sqrt{h^{2}+k^{2}+l^{2}}$$

where *h*, *k*, and *l* are the Miller indices of the Bragg diffraction plane.

**Figure 2:**
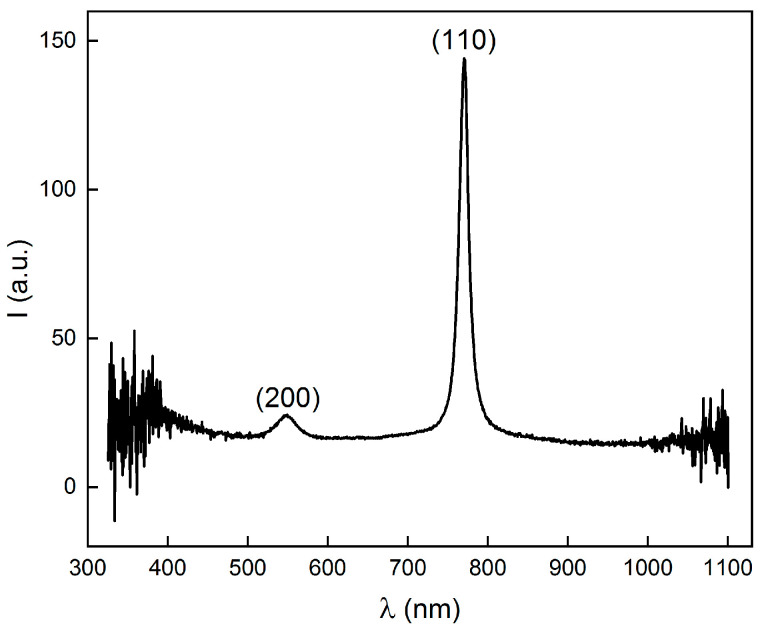
Reflection spectrum of the colloidal crystals formed in an aqueous solution.

A typical spectrum of the colloidal crystals is shown in Figure 2. The sharp main peak around 770 nm indicates the presence of a crystal lattice. The wave vectors of the two peaks are 21.7 μm^−1^ and 30.5 μm^−1^, and the ratio is $1:\sqrt{2}$. Therefore, the lattice type was a body-centered cubic (bcc), with the two peaks attributed to (110) and (200), respectively [40]. Moreover, the full width at half maximum (FWHM) has a close relationship with the quality of the crystals. A narrower FWHM means a higher crystal quality and fewer crystal defects, as imperfection within the crystals tends to broaden the FWHM. Here, the main peak (100) was very sharp and narrow, indicating the defects in the colloidal crystals were at a low level.

The experiments were conducted at 25 ± 2 °C in an air-conditioned room. In a typical procedure, the liquid colloidal crystals that were formed in a salt-free condition were transferred to the sample tube, and then one of the Dialysis Units was inserted into the tube, followed by the addition of a salt solution with the desired concentration. Special attention should be paid to make sure that there is gentle but sufficient contact between the colloidal crystals and the salt solution. Because of the low MWCO of the membrane, the colloidal particles cannot pass the membrane into the salt solution, but salt can diffuse into the crystal region. The connection between the sample tube and the Dialysis Units was sealed with laboratory film (PARAFILM, Chicago, IL, USA) to avoid both contamination with airborne CO_2_ and the evaporation of water. Through diffusion, the salt gradients ($\nabla C_{s}$) were established gradually throughout the sample tube, and the lattice constant variations induced by the salt diffusion were measured in situ by a reflection spectroscopy. The beam size was about 1 mm, and the penetration depth was basically near the wall. To check whether there was any temperature change in the vicinity of the beam, a temperature detector was placed at the optical fiber head. It was found that the temperature variation was smaller than 0.5 °C within two hours, so the heating effect on the sample can be ignored.

In addition, the influence of the dimensions of the sample tube on the experiment must be evaluated. It has been reported that too small of a tube radius would induce fluctuations in the crystallization process, but these fluctuations quickly diminished as the radius of the tube increased. For a tube radius exceeding 2 mm, the fluctuations can be ignored completely [41]. On the other hand, the average crystallite’s size *L* can be estimated from the width of the peak (110) with the Scherrer equation L=2πK/∆q, where *K* = 1.155 is the Scherrer constant for a crystal of cubic shape. The calculated *L* was approximately 20 μm, which was roughly two orders of magnitude smaller than the dimensions of the sample tube and the Dialysis Unit. Therefore, it can be inferred that the dimensions should have no influence on the experimental results.

## 3. Results and Discussion

**Figure 3:**
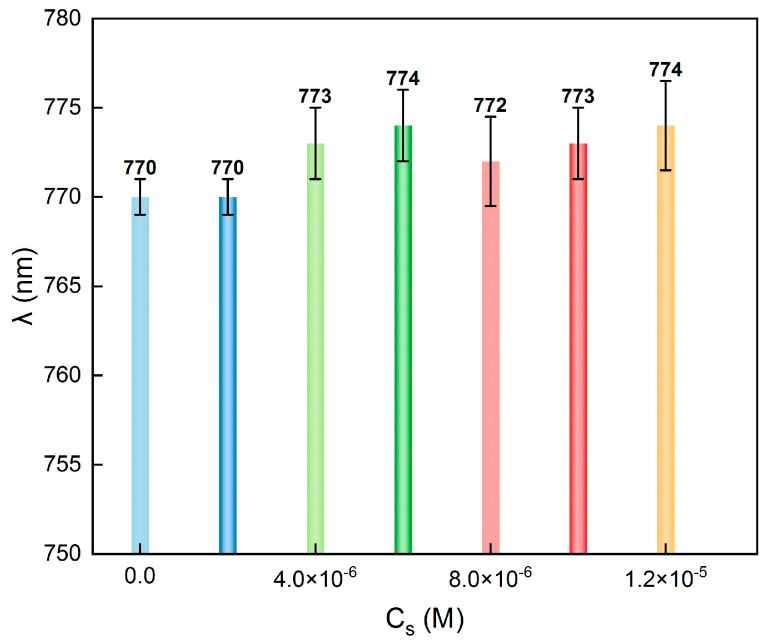
Peak wavelength of colloidal crystals formed in different and homogeneous *C*_s_.

In general, the colloidal particles can self-assemble to form colloidal crystals in concentrations of low electrolytes (*C*_s_), due to a strong interparticle repulsion. With the increase in *C*_s_, the repulsion becomes weaker, and the crystals will melt when the scale of repulsion is comparable to the thermal fluctuation. Nevertheless, within a specific range of moderate electrolyte concentration, colloidal crystals are able to persist. We prepared colloidal crystals with the same volume fraction in different but homogeneous *C*_s_. The reflection peak wavelengths are shown in Figure 3. It can be seen that the primary peak wavelengths were all located at approximately 770 nm, although the Debye length *λ*_*D*_ (∝*C*_s_^−1/2^$\propto {C_{s}}^{-1/2}$) decreased one order of magnitude from pure water to 1.2 × 10^−5^ M (the critical melting salt concentration). Clearly, the crystal lattice was maintained and basically unchanged when the *C*_s_ were homogeneous.

**Figure 4:**
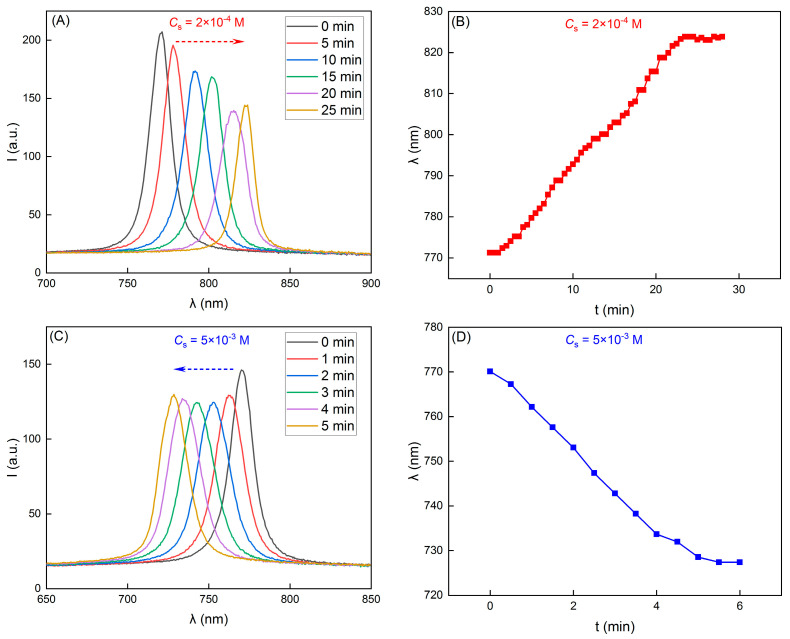
Reflection spectra and the corresponding peak wavelengths of the colloidal crystals during the diffusion of the salt solution with different concentrations. (**A**,**B**) *C_s_* = 2 × 10^−4^ M, and (**C**,**D**) *C_s_* = 5 × 10^−3^ M.

However, it became completely different when the electrolytes were not homogeneous; that is to say, a salt concentration gradient existed. In Figure 4, we can see the variations of the reflection spectra and the peak wavelengths of colloidal crystals on the salt gradient. Here, the gradient was introduced by adding a NaCl solution in the upper part of the sample cell (refer to Figure 1). Under this condition, the crystal lattice changed. Of particular interest is the observation that the variations of *λ* reversed when the *C*_s_ were increased from 2 × 10^−4^ M to 5 × 10^−3^ M. Specifically, when the NaCl solution in the upper part was 2 × 10^−4^ M, the colloidal crystals lattice expanded, resulting in a shift of λ from an initial 770 nm to 823 nm within 25 min (see Figure 4A,B), a typical red shift in the stop band. However, when the concentration of the NaCl solution was increased to 5 × 10^−3^ M, the crystal lattice was compressed and *λ* changed from the initial 770 nm to 728 nm in 5 mins (Figure 4C,D), a typical blue shift in the stop band. As shown in Figure 3, the differences in *C*_s_ cannot change the reflection peak wavelength *λ*. Therefore, the observed variations of *λ* here were most likely attributable to the top–down diffusion of the NaCl solution and the resulting salt gradient $\nabla C_{s}$.

**Figure 5:**
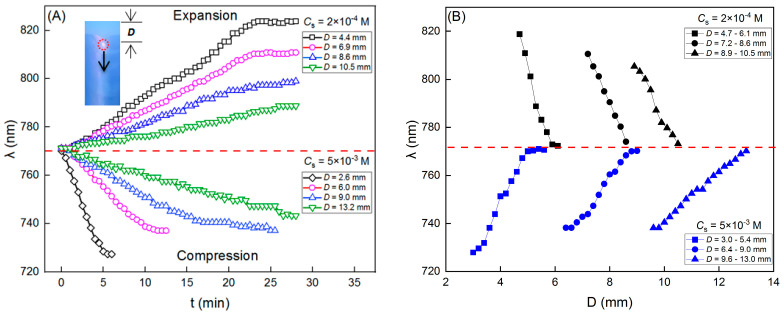
(**A**) Crystal lattice expansion and compression induced by salt diffusion. Inset: schematic illustration of the measurements. (**B**) Reflection wavelength evolutions from the measuring point to the bulk of colloidal crystals.

To further investigate the evolution of crystal lattices along the direction of salt diffusion, we measured the changing tendency of *λ* at four points with a different *D* (the distance between the measuring point and the membrane). The results obtained at each of the *C*_s_ are shown in Figure 5A. It should be mentioned that when the front of the salt diffusion reached the critical melting concentration (10^−5^ M), the crystals melted, and the corresponding measurement was stopped. Then, the head of the optical fiber was moved downward in a 0.5 mm step; the *λ* evolutions in the moving process are shown in Figure 5B. The next measurement was conducted at the point where the reflection peak wavelength returned to the initial *λ*_0_. The experimental results suggested that the changing tendency of *λ* was similar at a different *D*: for *C*_s_ = 2 × 10^−4^ M, *λ* increased, meaning crystal lattice expansion, while for *C*_s_ = 5 × 10^−3^ M, *λ* decreased, implying crystal lattice compression. However, the range of the reflection peak variation $∆\lambda =\lambda -\lambda_{0}$ became smaller when the measuring point was away from the membrane. The maximum ∆λ was +54 nm and −43 nm for 2 × 10^−4^ M and 5 × 10^−3^ M, respectively, demonstrating similarities to the wavelength variations induced by electrical or mechanical factors as reported in previous studies [11,42]. Additionally, from Equation (2) the deformations of lattice constant $∆L_{a}=L_{a}-L_{a,0}$ and strain $\varepsilon =∆L_{a}/L_{a,0}$ were calculated (listed in Table 1), which confirmed the expansion and compression of the crystal lattice. Finally, the number density of the local particles or the volume fraction in the crystalline region can be evaluated by $\phi =\frac{4\pi ({2a)}^{3}}{3\sqrt{2}{\left(\lambda /\nu \right)}^{3}}$, where *a* is the particle radius. Consequently, an increase in *λ* corresponds to a decrease in  ϕ, indicating that particles have migrated away from the region under observation, and vice versa. Now, where exactly did these particles go? Let us consider the case of *D* = 4.4 mm in *C*_s_ = 2 × 10^−4^ M (Figure 5A). At this point, *λ* increased, and so the number of particles decreased. In the lower part of this point, denoted as *D* = 4.7–6.1 mm (Figure 5B), *λ* was also larger than the initial *λ*_0_, suggesting a decrease in the particles within this region. Therefore, considering the combined *λ* variations both in the measuring region and the lower vicinity, we deduced that particles moved upward to the membrane, although particle concentrations were not obtained in the upper liquid part. For the higher salt concentration of *C*_s_ = 5 × 10^−3^ M, the changing tendency of *λ* and the direction of particles migration were reversed. Therefore, in addition to the routinely used lattice deformation and stop band regulation methods such as electric field and mechanical loading, we here demonstrated an alternative approach by utilizing salt gradients.

From the above discussions, it is obvious that the salt gradient plays a significant role in the lattice deformation of colloidal crystals. In fact, the spontaneous migration of particles in salt concentration gradients can be described by the classical theory of diffusiophoresis, which generally contains two contributions named chemiphoresis (CP) and electrophoresis (EP). CP arises due to the pressure imbalance inside the electrical double layer (EDL) near a charged surface. In the higher salt concentration, the pressure at the side is larger than that on the lower side, which drives a diffusioosmotic slip flow down the salt gradient. The particles move in the opposite direction, and it is always upward along the salt gradient. On the other hand, EP is in a manner similar to chemiphoresis; however, by applying an external electric field, cations and anions with different diffusivities in the solution can cause a self-generated electric field spontaneously. The particles having a zeta potential or a surface charge move in this electric field, and the direction depends on the signs of the surface charge and electric field. Theoretically, the DP velocity of the particles in the electrolyte concentration gradients is given by $v_{p}=\Gamma_{p}\nabla lnC_{s}$, where $\Gamma_{p}$ is the diffusiophoretic mobility in the binary 1:1 electrolyte gradient.

(3) $$\Gamma_{p}=\frac{\varepsilon k_{B}T}{\eta e}\;\left(\zeta_{p}\beta +\frac{4k_{B}T}{e}ln\left(cosh\frac{e\zeta_{p}}{4k_{B}T}\right)\right)$$

Here, ε and η are the permittivity and viscosity of the solution, $k_{B}T$ is the thermal energy, *e* is the elementary electric charge, $\zeta_{p}$ is he zeta potential of the particle, and $\beta ={(D}_{+}-D_{-})/{(D}_{+}+D_{-})$, which is a parameter describing the difference of diffusion coefficients of cations ($D_{+}$) and anions ($D_{-}$). The first and second items in Equation (3) are the contributions of EP and CP, respectively.

For the NaCl used here, β = −0.21, meaning that anions are the faster ions and the diffusion potential is downward, so negatively charged particles should move upward. Because CP is always upward, the particles should move upward and lattice expansion is expected, and this was indeed observed for the smaller *C*_s_. It is, however, not the case for the higher *C*_s_, where the particles reversed their direction and lattice compression happened. It seemed that, by solely considering the DP effect, we cannot give a satisfactory explanation of the experimental results.

**Figure 6:**
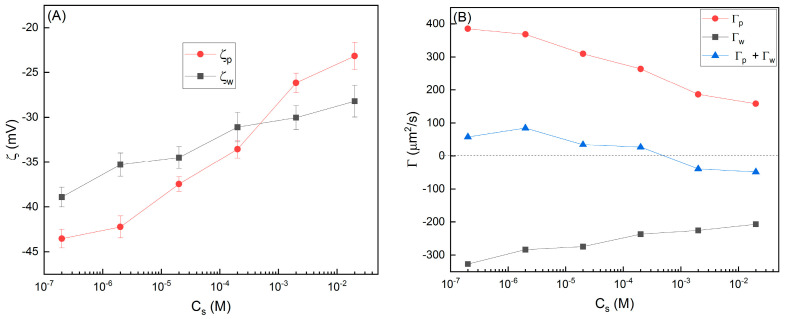
(**A**) The zeta potentials of particles ($\zeta_{p}$) and wall ($\zeta_{w}$) in different *C*_s_. (**B**) The calculated mobility of particle DP ($\Gamma_{p}$), wall DO ($\Gamma_{w}$), and their summations with varied *C*_s_.

Practically, in addition to the zeta potential of particles $\zeta_{p}$, when the solid wall contacted the liquid, it also generated electrical potential $\zeta_{w}$ at the solid–liquid interface. The charged wall induced DO flow in the same manner as the DP of the particles, but in the opposite direction. The mobility of the DO flow $\Gamma_{w}$ depends on $\zeta_{w}$ and $\nabla C_{s}$. Assuming wall and particles were subjected to an identical solution environment, $\nabla C_{s}$ was assumed to be equal. As a result, the motion of the particles near the wall was determined by the zeta potential of both the wall and the particles. The zeta potential is usually treated as a constant in most DP experiments, but some recent studies showed that the zeta potential also varies with the properties of the solution. In order to explore the underlying mechanism of lattice expansion and expression, we measured the variations of $\zeta_{w}$ and $\zeta_{p}$ in different *C*_s_, and the results are shown in Figure 6A. Firstly, we found both $\zeta_{w}$ and $\zeta_{p}$ were negative. The transition of $\zeta_{p}$ from negative to positive, a phenomenon typically associated with the reversal of particle migration direction, was not observed. Secondly, due to EDL compression, both $\zeta_{w}$ and $\zeta_{p}$ decreased when the *C*_s_ was increased. However, $\zeta_{w}$ changed more smoothly than $\zeta_{p}$, which possibly stems from the surface modification of polypropylene during the fabrication process [43,44,45,46]. Thirdly, a cross point of $\zeta_{w}$ and $\zeta_{p}$ existed in the *C*_s_ range around 10^−3^–10^−4^ M. For *C*_s_ below 10^−4^ M, $\zeta_{p}\;$(as an absolute value) was larger than $\zeta_{w}$. If *C*_s_ increased above 10^−3^ M, $\zeta_{w}$ was larger than $\zeta_{p}$. Coincidently, the transition point of lattice expansion and compression was also in this range of *C*_s_.

Furthermore, we calculated the mobility of particle DP ($\Gamma_{p}$) and wall DO flow ($\Gamma_{w}$), as well as the summation of $\Gamma_{p}$ and $\Gamma_{w}$ in Figure 6B. $\Gamma_{p}$ were all positive and $\Gamma_{w}$ were all negative, but their summation changed from positive to negative. Apparently, the competition between particle DP and wall DO flow determined the direction of particle migration and the resultant lattice expansion and expression. In the lower *C*_s_ range, the particle DP effect dominates and the summation of mobility is positive, so the particles migrated upward close to the membrane and the crystal lattice expanded. Conversely, in the higher *C*_s_ range, the DO flow of the tube wall dominated, and the summation of mobility changed to negative, so the particles migrated downward away from the membrane and the crystal lattice was compressed. To make a short summary, due to $\zeta_{p}$ and $\zeta_{w}$ having different responses to the salt concentrations, changes in the salt concentrations result in a crossover in their zeta potentials. The value of the zeta potential dictates the related strengths of diffusiophoresis and diffusioosmosis, and the summation of them determines the migration of colloidal particles. In low salt concentrations, the zeta potential of the particle is larger (as an absolute value), and upward particle diffusiophoresis dominated; in high salt concentrations, the zeta potential of the wall is larger, and downward wall diffusioosmosis dominated. The migration of particles changed the particles’ density, so the peak wavelength of the liquid colloidal crystals could be altered in a convenient manner. This proposed strategy, in principle, could be implemented to other systems needing to sort charged microscale objects suspended in solution.

**Figure 7:**
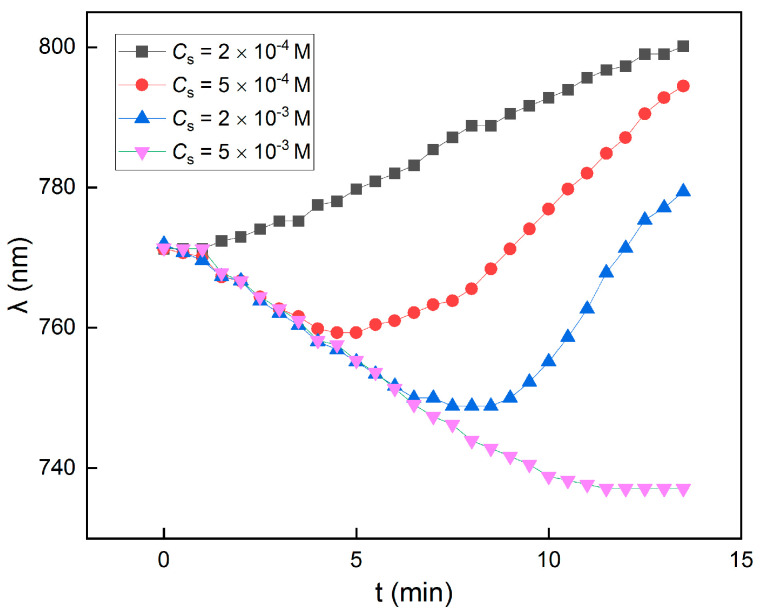
Peak wavelength variations in different *C*_s_. Under low and high *C*_s_ (2 × 10^−4^ M, 5 × 10^−3^ M), the variations are monotonous, while in moderate *C*_s_ (5 × 10^−4^ M, 2 × 10^−3^ M), the variations transitioned from compression to expansion.

Due to the diffusion of the salt ion, the salt gradient may decrease over time. Therefore, based on the above-mentioned mechanism, a transition from expansion to compression can be naturally anticipated if the salt concentration is not high enough. To further verify this conjecture and the above-mentioned mechanism, we included two additional sets of data, 5 × 10^−4^ M and 2 × 10^−3^ M, for the lattice variations. The results are shown in Figure 7. Obviously, under moderate *C_s_* (5 × 10^−4^ M, 2 × 10^−3^ M) around the crossover in Figure 6A, the change tendencies of the lattice with time were different from those under low (2 × 10^−4^ M) and high (5 × 10^−3^ M) *C_s_*. The lattice variations were not monotonous; instead, the crystal lattice was first compressed and then expanded, until under high *C_s_* (5 × 10^−3^ M) the lattice expansion was completely suppressed and only lattice compression was observed. This result further validated the mechanism described in previous paragraphs.

## 4. Conclusions

In this study, we demonstrated that the expansion or compression of colloidal crystal lattices can be controlled by simply adjusting the concentration of the salt solution in contact with the crystals. Through altering the salt concentration, we were able to tune the reflection peak wavelength of the crystals from +54 nm to −43 nm compared to the original peak wavelength. It is evident that the reversal in the direction of the particle migration cannot be explained solely by the common diffusiophoresis of particles in the salt gradient, because variations of salt concentrations did not alter the direction of the salt gradient. Therefore, the diffusioosmotic fluid flow induced by the charged container wall must be taken into account. Additionally, although both the zeta potentials of the particles and the wall decreased with the increasing salt concentrations, their changing tendencies were not identical. The zeta potential of the tube wall changed more slowly and smoothly than that of the colloidal particles. Consequently, in low salt concentrations, particle diffusiophoresis dominated, while wall diffusioosmosis was predominant in higher salt concentrations. The competition between these two mechanisms dictated the direction of particle migration and the resultant expansion or compression of the crystal lattices. Based on our experimental findings and theoretical analysis, we believe that it is possible to further optimize the tuning of the wavelength of colloidal crystals using diffusiophoresis. Moreover, this general approach may also find applications in protein separation and purification.

## Figures and Tables

**Table 1:** Variations of ∆λ, $∆L_{a}$, and ε with *D* in different *C*_s_.

*C_s_* (M)	*D* (mm)	∆λ (nm)	$∆L_{a}$ (nm)	ε
2 × 10^−4^	4.4	54	29	0.07
	6.9	41	22	0.05
	8.6	28	15	0.04
	10.5	19	10	0.02
5 × 10^−3^	2.6	−43	−23	−0.06
	6.0	−33	−17	−0.04
	9.0	−31	−16	−0.04
	13.2	−27	−14	−0.03

## Data Availability

The data that support the findings of this study are available from the corresponding author upon reasonable request.
